# The Pin1–cis P-tau Axis: A Key Common Early Pathogenic Driver, Biomarker and Therapeutic Target Across Neurodegenerative, Traumatic, and Vascular Cognitive Disorders

**DOI:** 10.3390/cells15181650

**Published:** 2026-09-12

**Authors:** Zhixiong Li, Yitong Li, Rui Liu, Yuanming Michelle Zhu, Ruizhi Wang, Kun Ping Lu, Xiao Zhen Zhou

**Affiliations:** 1 Departments of Biochemistry and Oncology, Schulich School of Medicine & Dentistry, Western University, London, ON N6A 5C1, Canada; zli3273@uwo.ca (Z.L.);; 2 Robarts Research Institute, Western University, London, ON N6A 5B7, Canada; 3 Department of Physiology and Pharmacology, Schulich School of Medicine & Dentistry, Western University, London, ON N6A 5C1, Canada; 4 Department of Pathology and Laboratory Medicine, Schulich School of Medicine & Dentistry, Western University, London, ON N6A 5C1, Canada; 5 Lawson Health Research Institute, Western University, London, ON N6C 2R5, Canada

**Keywords:** Pin1, cis P-tau, cistauosis, Alzheimer’s disease, traumatic brain injury, ischemic stroke, vascular cognitive disorders, preeclampsia, biomarker, neurodegeneration

## Abstract

Tauopathies comprise a heterogeneous group of neurological and systemic disorders characterized by pathological tau dysregulation, traditionally including Alzheimer’s disease (AD), traumatic brain injury (TBI), and chronic traumatic encephalopathy (CTE). Emerging evidence suggests that tauopathy may also occur in selected vascular and systemic disease contexts, including ischemic stroke, vascular dementia (VaD), and preeclampsia (PE), although the strength and clinical significance of this evidence vary among these conditions. Compelling evidence suggests that the Pin1-cis phosphorylated tau (cis P-tau) axis may represent a convergent molecular mechanism linking these conditions. Pin1 is a phosphorylation-specific peptidyl-prolyl cis-trans isomerase that protects against tauopathy and cognitive impairment by catalyzing conversion of pathological cis P-tau to the physiological trans conformation at phosphorylated Thr231-Pro motifs. Under stress conditions such as oxidative stress, hypoxia, inflammation, mechanical injury, or aging, Pin1 activity becomes impaired through oxidation, phosphorylation, sequestration, or transcriptional downregulation, resulting in the accumulation of neurotoxic cis P-tau. cis P-tau is proposed to initiate a pathogenic cascade termed cistauosis in neurons, characterized by axonal microtubule collapse and transport failure, mitochondrial dysfunction, synaptic degeneration, neuroinflammation, neuronal death, and prion-like spread. Importantly, cis P-tau appears rapidly after insults long before overt neurofibrillary tangle (NFT) formation in several experimental and clinical contexts, supporting its role as an upstream pathogenic driver and early biomarker. Crucially, conformation-specific monoclonal antibodies targeting cis P-tau selectively eliminate pathological cis P-tau while sparing physiological trans P-tau, demonstrating promising therapeutic effects in preclinical models of TBI, vascular injury, AD-related tauopathy, and PE. This review summarizes the molecular basis of the Pin1–cis P-tau axis, examines its role across conventional and unconventional tauopathies, evaluates its biomarker potential, and highlights cis-specific immunotherapy as a potentially broad therapeutic strategy for neurodegenerative, traumatic, and vascular cognitive disorders.

## 1. Introduction

Tauopathies are a broad family of disorders characterized by pathological tau phosphorylation, conformational alteration, aggregation, and progressive neurodegeneration [1,2]. Among them, Alzheimer’s disease (AD) is the most prevalent neurodegenerative dementia and currently affects more than 55 million individuals worldwide, with incidence expected to rise dramatically due to population aging [3,4]. Beyond AD, traumatic brain injury (TBI) and chronic traumatic encephalopathy (CTE) are increasingly recognized as major contributors to long-term cognitive impairment and dementia development [5,6,7]. In parallel, ischemic stroke and vascular dementia (VaD) have been strongly linked to post-stroke cognitive decline and vascular contributions to dementia [8,9], while preeclampsia (PE) has recently been associated with increased long-term risk of neurodegenerative disorders and tau-related pathogenic mechanisms [10,11]. Despite major clinical differences among these disorders, converging studies suggest that some shared molecular mechanisms may contribute to early cellular and neuronal dysfunction before irreversible pathology occurs [12,13,14].

Historically, tauopathy research has primarily focused on late-stage pathological hallmarks, particularly neurofibrillary tangles (NFTs) composed of aggregated hyperphosphorylated tau [15,16]. However, traditional therapeutic approaches targeting NFTs or amyloid-β plaques have produced only limited clinical benefits [17,18,19]. Recently, increasing evidence indicates that soluble tau species, rather than mature fibrillar aggregates, may represent the principal neurotoxic species responsible for early neuronal injury [20,21,22]. Among these, phosphorylated tau at Thr231-Pro undergoes a unique conformational switch between cis and trans isomers, creating functionally distinct tau species [23,24]. This discovery substantially advanced the current understanding of tau biology and suggested a potential upstream mechanism contributing to neurodegeneration.

Under physiological conditions, the peptidyl-prolyl cis-trans isomerase NIMA-interacting 1 (Pin1) helps maintain tau conformational homeostasis to protect against tauopathy and neurodegeneration by regulating the cis/trans switch of phosphorylated tau [24,25]. Under disease-associated stress, Pin1 activity may become impaired, permitting accumulation of pathogenic cis P-tau [14,26]. Consequently, cis P-tau has been proposed as an early neurotoxic conformer that links diverse upstream insults to axonal dysfunction, mitochondrial impairment, synaptic degeneration, neuroinflammation, and pathological tau propagation [12,13,14]. Accumulating evidence further suggests that dysregulation of the Pin1–cis P-tau axis may represent a conserved mechanism shared across both conventional and unconventional tauopathies, thereby providing a unifying framework for understanding early neurodegenerative processes [27,28].

This review comprehensively summarizes the molecular basis of the Pin1–cis P-tau axis and its involvement across both conventional and unconventional tauopathies. We first discuss the structural and functional regulation of Pin1 and the cis/trans conformational switch of tau, and then review current evidence implicating this pathway in AD, TBI/CTE, ischemic stroke and vascular dementia, as well as PE. Because the strength and nature of the supporting evidence differ substantially among these conditions, we distinguish findings derived from human samples, experimental disease models, and clinical studies throughout the review. Mechanistic and interventional evidence is strongest in experimental models of AD-related tauopathy, TBI and vascular models. In ischemic stroke, much of the mechanistic and therapeutic evidence remains preclinical, while in PE, although circulating and placental cis P-tau has been shown to play a major role in the acute disease process, its relationship to long-term maternal dementia remains to be established. Finally, we evaluate the translational potential of cis P-tau as a shared biomarker and therapeutic target and discuss future directions for precision-medicine approaches to early disease detection and intervention.

This review provides a narrative synthesis of the literature on the Pin1–cis P-tau axis. We prioritized primary studies directly evaluating Pin1, conformation-specific cis P-tau, or cis P-tau-targeted interventions across the disease contexts discussed, together with relevant studies addressing tau biology, biomarkers, and therapeutic approaches. Reference lists of key primary studies and reviews were also examined to identify additional relevant publications. Because this is a narrative rather than systematic review, the literature was selected to provide a representative and critical overview of the field rather than an exhaustive or quantitatively weighted analysis.

## 2. Molecular Basis of the Pin1–cis P-tau Axis

### 2.1. Pin1: Structure, Function, and Regulation in Tau Homeostasis

Pin1 is a highly conserved and unique phosphorylation-specific peptidyl-prolyl cis-trans isomerase (PPIase) that catalyzes the conformational change between cis and trans P-tau. Structurally, Pin1 has two major functional domains: an N-terminal WW domain and a C-terminal catalytic PPIase domain [29,30].

**Figure 1:**
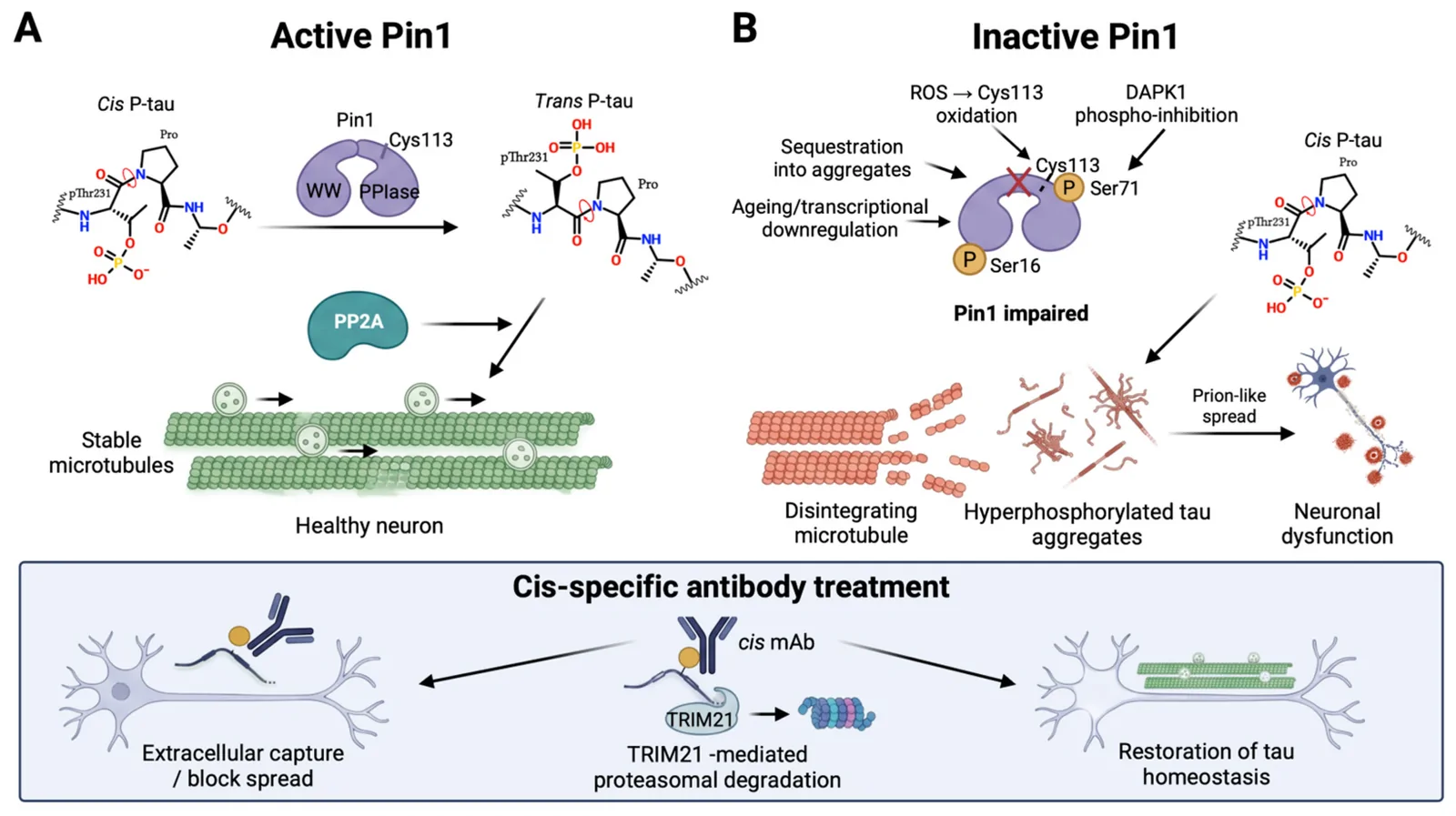
The Pin1–cis P-tau conformational switch in healthy and dysfunctional neurons. Phosphorylation of tau at Thr231 generates a proline-directed motif that can adopt two distinct conformations. (**A**) In the healthy neuron, the peptidyl-prolyl isomerase Pin1 engages pThr231-Pro through its WW domain and, via its PPIase domain, converts the pathological cis conformation to the physiological trans form. trans P-tau is accessible to protein phosphatase 2A (PP2A), supporting normal dephosphorylation, microtubule stability and axonal transport. (**B**) Oxidative modification of Cys113, inhibitory phosphorylation by DAPK1, sequestration into aggregates, and age-related transcriptional decline each inactivate Pin1. The pThr231-Pro motif then remains locked in the cis conformation; cis P-tau resists PP2A, loses microtubule affinity and propagates to neighboring neurons in a prion-like manner. A conformation-specific monoclonal antibody (cis mAb) binds cis P-tau selectively and clears it through extracellular neutralization and tripartite motif-containing protein 21 (TRIM21)-mediated proteasomal degradation, while sparing trans P-tau.

The WW domain mediates selective substrate recognition by binding to certain phosphorylated Ser/Thr-Pro motifs, including the pThr231-Pro motif in tau [30,31], whose isomerization is significantly slowed down upon phosphorylation and cannot be catalyzed by other PPIases [32]. After substrate binding, the catalytic PPIase domain induces cis-trans isomerization of the peptide bond preceding proline residues [29,33]. Importantly, the catalytic cysteine residue Cys113 is essential for Pin1 enzymatic activity, and oxidative modification of this residue profoundly impairs Pin1 function (Figure 1) [26].

Pin1 substrates participate in numerous cellular processes, including cell cycle regulation, apoptosis, transcription, inflammation, and neuronal function [29,30]. Notably, Pin1 specifically recognizes phosphorylated Ser/Thr-Pro motifs and regulates protein conformation, stability, localization, and downstream signaling [29,32].

In the nervous system, tau is among the most pathologically relevant Pin1 substrates [31,33]. Tau normally functions as a microtubule-associated protein that regulates microtubule dynamics and supports axonal transport [2,34,35,36]. Proper tau function depends not only on phosphorylation status but also on conformational structure [24,31]. Following phosphorylation at Thr231, tau can adopt either cis or trans conformations [24,33]. The spontaneous interconversion rate between these conformations is extremely slow under physiological conditions, making enzymatic catalysis necessary for efficient regulation [30,32]. Pin1 can significantly accelerate the cis-trans isomerization of pThr231-tau and promote the conversion of pathological cis P-tau into physiological trans P-tau, thereby promoting microtubule-associated function, tau dephosphorylation and degradation [31].

Consequently, Pin1 possesses potent neuroprotective properties in part through its regulation of tau [31,33]. Genetic deletion of Pin1 in mice induces progressive tau hyperphosphorylation, neurodegeneration, synaptic dysfunction, and age-dependent cognitive impairment [37,38]. Conversely, preserved Pin1 activity has been associated with reduced tau pathology and improved neuronal function [38,39]. These findings support a central role for Pin1 in maintaining neuronal homeostasis, including the regulation of tau. However, because Pin1 regulates other substrates involved in amyloid precursor protein (APP) processing, transcription, apoptosis, inflammatory signaling, and other cellular processes, phenotypes resulting from global Pin1 deficiency cannot be attributed exclusively to altered tau isomerization or cis P-tau accumulation.

### 2.2. cis vs. trans P-tau: Conformational Switch and Pathological Consequences

The tau protein is encoded by the *MAPT* gene on chromosome 17q21 and exists as six major isoforms in the adult human brain [40,41]. Physiologically, tau regulates microtubule dynamics and supports axonal transport [2,34].

Phosphorylation is a major regulatory mechanism controlling tau function [42]. However, phosphorylation alone does not fully determine tau pathogenicity. Instead, phosphorylation at Thr231-Pro creates two structurally distinct conformations: cis and trans [24,43]. Despite having the same primary structure and phosphorylation modification, the cis and trans conformations exhibit significantly different functions and pathogenic characteristics (Figure 1 and Table 1) [24].

In particular, cis and trans P-tau differ drastically in microtubule affinity, dephosphorylation and degradation susceptibility, aggregation tendency, and neurotoxicity [14,24,43]. trans P-tau retains strong microtubule binding affinity and supports cytoskeletal stability. Structural analyses demonstrate that the trans conformation preserves proper orientation of tau microtubule-binding domains, enabling efficient interaction with tubulin polymers [24,31]. In contrast, the cis conformation disrupts these interactions and destabilizes microtubules [14,31].

Mounting evidence suggests that cis P-tau may be more directly involved in early neurotoxic processes than total phosphorylated tau protein or mature NFTs and should be considered as an important early pathogenic tau conformation driving neurodegeneration [13,14,24,43].

#### 2.2.1. Physiological trans P-tau

trans P-tau represents the physiological conformation of phosphorylated tau and performs essential neuronal functions [24,43]. trans P-tau maintains high affinity for neuronal microtubules and preserves cytoskeletal stability, thereby supporting normal tau function and microtubule integrity [14,31]. Stable microtubules are required for the transport of mitochondria, synaptic vesicles, and signaling molecules, and thus contribute to neuronal connectivity and synaptic function [45].

Under physiological conditions, Pin1-mediated conversion of cis to trans P-tau facilitates dephosphorylation by protein phosphatase 2A (PP2A), the major tau phosphatase in the brain (Figure 1A) [33,46]. Because PP2A preferentially recognizes the trans pSer/Thr-Pro conformation, trans P-tau undergoes efficient dephosphorylation and turnover, thereby preventing pathological accumulation and maintaining tau homeostasis [24,33]. Pin1-mediated conversion to the trans conformation therefore serves as a quality-control mechanism that preserves physiological tau function and limits the generation of pathogenic tau species [23,24].

Importantly, trans P-tau remains largely non-toxic and does not induce significant neurodegenerative changes [12,14]. Experimental depletion of trans P-tau from cultured neurons disrupts axonal microtubules and transport and promotes neurotoxicity, in contrast to cis P-tau [14].

These findings suggest that phosphorylation at Thr231 is not inherently pathogenic in the presence of intact functional Pin1 activity and that maintenance of the trans conformation is essential for neuronal homeostasis.

#### 2.2.2. Pathological cis P-tau

cis P-tau is considered a pathogenic tau conformer that contributes to early neurodegenerative processes. The term “cistauosis” has been coined to describe this early pathogenic cascade initiated by the accumulation of cis P-tau and characterized by loss of normal function and gain of toxic function that disrupt neuronal homeostasis [14,44]. Unlike trans P-tau, cis P-tau not only loses normal tau function, but also exhibits toxic gain-of-function properties that disrupt neuronal homeostasis, promote tau accumulation, and drive downstream neurodegenerative changes [12,14,24].

Mechanistically, cis P-tau shows reduced microtubule-binding capacity and impaired physiological tau function compared with the trans conformation [24,31,43]. This conformational shift may compromise tau-mediated microtubule stabilization, thereby contributing to cytoskeletal instability, axonal dysfunction, and impaired intracellular transport [24,47,48]. Because intact microtubules are essential for axonal and mitochondrial transport, cis P-tau-associated microtubule disruption may further promote local energy failure, synaptic dysfunction, and neuronal vulnerability [45,49,50,51].

cis P-tau is also less efficiently dephosphorylated by PP2A because PP2A preferentially recognizes the trans pSer/Thr-Pro conformation [33]. As a result, cis P-tau may progressively accumulate and escape normal tau homeostatic regulation, favoring the formation of soluble pathogenic tau species and later aggregation-associated pathology [23,24,33].

Another proposed pathological feature of cis P-tau is its ability to promote toxic tau propagation. Experimental studies suggest that cis P-tau may behave as a prion-like toxic conformer and contribute to tau-related pathology in recipient neurons [12,14,25]. More broadly, tau propagation may involve exosome-mediated release, synaptic transmission, and uptake by neighboring neurons, mechanisms that could contribute to the stereotypical spread of tau pathology across anatomically connected brain networks [52,53,54,55,56]. These findings support the view that cis P-tau has greater pathogenic and propagation potential than the trans conformation.

Pathologically, cis P-tau acts as an upstream toxic conformer that can trigger multiple downstream cascades, including mitochondrial impairment, oxidative stress, synaptic dysfunction, neuroinflammation, and neuronal loss [12,13,14]. In addition to its neuronal effects, cis P-tau-associated pathology may activate microglial and astrocytic inflammatory responses, further amplifying neuronal injury and disease progression [12,13]. Importantly, experimental administration of purified cis P-tau into wild-type, but not tau-null, mouse brains is sufficient to induce cistauosis, neurodegenerative changes and cognitive impairment even before mature NFT formation, supporting its role as an early pathogenic driver rather than a mere byproduct of late-stage tau aggregation [13,14,24].

### 2.3. Pin1 Dysregulation and Cistauosis: A Unifying Mechanism Across Tauopathies

**Figure 2:**
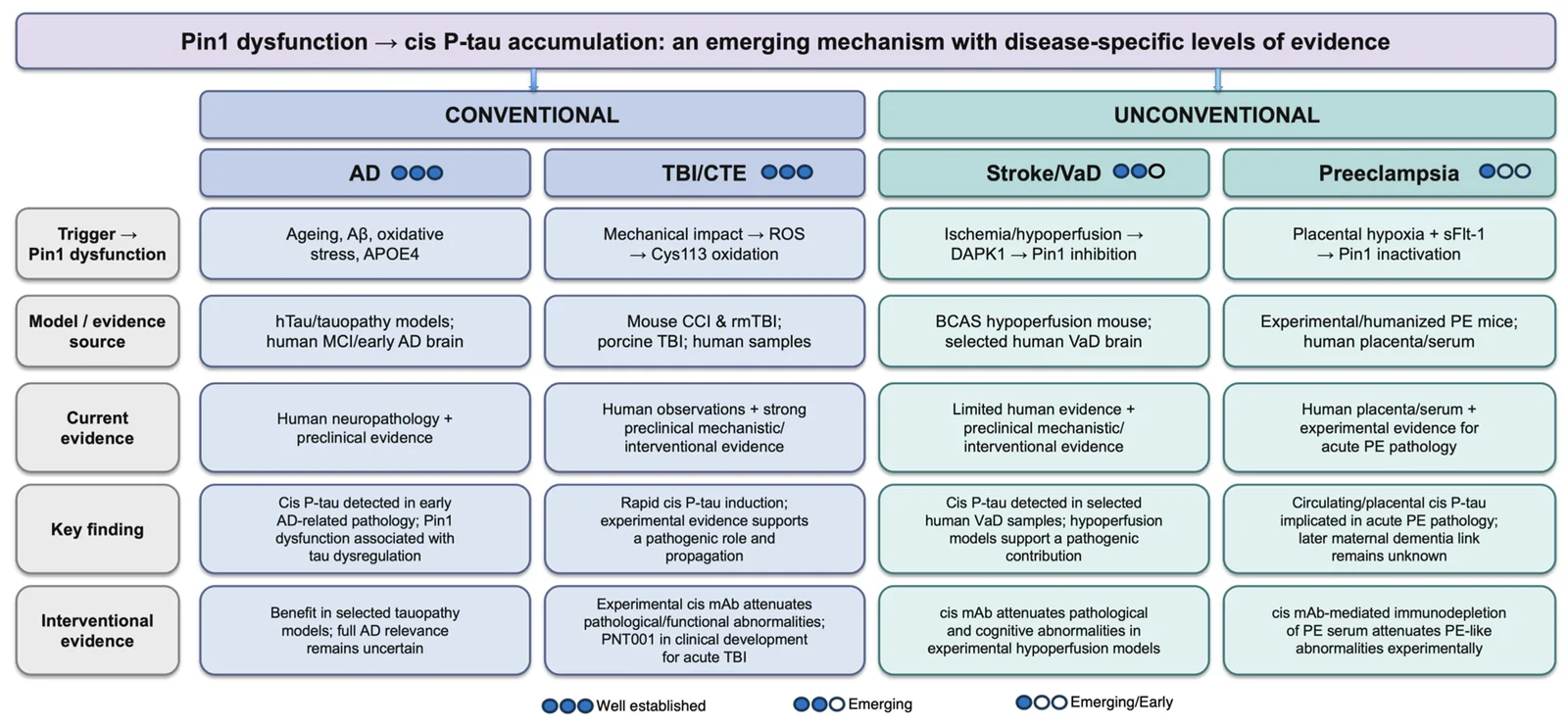
The Pin1–cis P-tau axis across conventional and unconventional tauopathies. Evidence type and strength differ substantially among disease contexts; columns are not intended to imply equivalent pathogenic or therapeutic evidence.

**Figure 3:**
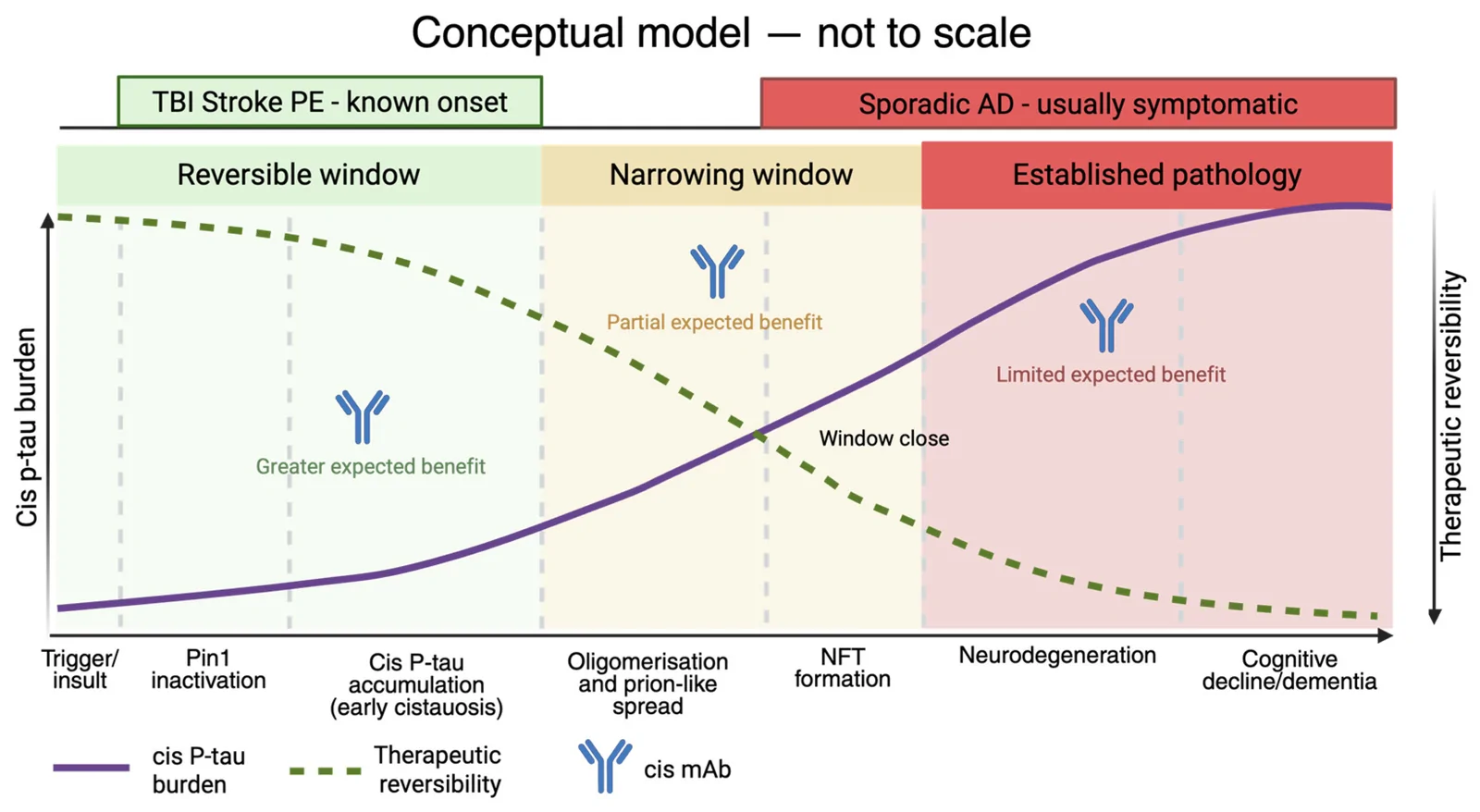
Conceptual model of the proposed temporal relationship between cis P-tau associated pathology and the potential closing therapeutic window. Following disease-associated triggers or insults, Pin1 inactivation may promote early cis P-tau accumulation, cistauosis and prion-like spread, eventually followed by oligomerization, aggregation, and NFT formation, and other established pathological changes. The schematic illustrates the model that earlier targeting of cis P-tau may provide greater therapeutic opportunity than intervention after substantial downstream pathology has developed. Importantly, the curves, stage boundaries, crossover point, and relative therapeutic responsiveness shown are conceptual and are not derived from quantitative experimental measurements or intended to define a specific therapeutic window. TBI, stroke, PE and sporadic AD have substantially different disease time courses; their placement in the figure is therefore illustrative and should not be interpreted as temporal equivalence across diseases. The timing and efficacy of cis P-tau-targeted intervention remain to be established in disease-specific clinical studies.

One of the major conceptual advances in modern neurodegeneration research is the emerging recognition that multiple neurological disorders may converge on shared pathogenic pathways involving Pin1 dysfunction and cis P-tau accumulation (Figure 2 and Figure 3) [13,14,27,28]. Traditionally, tauopathies were classified primarily as chronic neurodegenerative diseases characterized by mature tau aggregates, including AD, Pick’s disease, corticobasal degeneration, and progressive supranuclear palsy [57]. However, increasing evidence demonstrates that acute brain injury, vascular dysfunction, amyotrophic lateral sclerosis (ALS), and systemic inflammatory disorders can also induce pathological tau conformational changes [11,12,13,14,58].

The Pin1–cis P-tau axis provides a mechanistic framework linking these seemingly distinct disorders [27,59]. Diverse pathological conditions—encompassing conventional and unconventional tauopathies—converge on dysregulation of the Pin1–cis P-tau axis and subsequent activation of cistauosis-related pathways (Figure 2) [25,59].

Importantly, as a stress-responsive enzyme, Pin1 activity is precisely regulated by cellular redox state, phosphorylation status, and intracellular localization [26,30,60]. Under physiological conditions, this regulation ensures balanced tau turnover and prevents accumulation of toxic tau conformers in response to stress. However, excessive or persistent stresses or insults such as hypoxic or oxidative stress, injury, inflammation, and aging may progressively impair Pin1 activity, predisposing neurons to cis P-tau accumulation and neurodegeneration [26,30,38,61].

#### 2.3.1. Mechanisms of Pin1 Inactivation in Disease

A central feature shared across multiple tauopathies is pathological inactivation of Pin1, which may lead to accumulation of toxic cis P-tau and initiation of cistauosis [12,14,24]. Multiple disease-associated mechanisms impair Pin1 activity, including oxidative inhibition, sequestration by pathological tau, inhibitory phosphorylation, and transcriptional downregulation [26,62]. These mechanisms often coexist and synergistically amplify neuronal dysfunction.

##### Oxidative Inhibition

Oxidative stress is one of the major mechanisms of Pin1 dysfunction in neurodegenerative disease [61,63]. Reactive oxygen species (ROS), generated during aging, mitochondrial dysfunction, hypoxia, ischemia, or traumatic injury, oxidize the catalytic cysteine residue Cys113 within the Pin1 PPIase domain, disrupting substrate binding and abolishing isomerase activity [26,64].

In AD brains, oxidized Pin1 accumulates prominently in hippocampal and cortical neurons and correlates with both NFT burden and cognitive decline [26,61,65]. TBI can also lead to rapid Pin1 dysfunction and cis P-tau induction, while ROS generation typically peaks within hours of injury [12,66,67].

Importantly, oxidative inactivation creates a vicious cycle. Reduced Pin1 activity promotes cis P-tau accumulation, while cis P-tau itself induces mitochondrial dysfunction and further ROS production [14,28]. This feed-forward loop may amplify progressive neurodegenerative signaling.

##### Sequestration by cis P-tau

Pathological cis P-tau accumulation may be associated with redistribution or sequestration of Pin1 into insoluble tau aggregates, thereby reducing functional soluble Pin1 pools [24,68]. In AD brains, Pin1 colocalizes with hyperphosphorylated tau within NFTs and neuritic plaques [68]. Similar pathological cis P-tau accumulation and tau-related abnormalities have been observed in TBI and CTE-related pathology [12,14].

This mechanism establishes another pathogenic amplification loop: initial dysfunction of Pin1 leads to the accumulation of cis P-tau [14,24], while pathological cis P-tau accumulation may further contribute to Pin1 sequestration and disruption of cis-trans isomerization [68]. Over time, progressive sequestration severely compromises neuronal tau homeostasis.

Sequestration additionally disrupts nuclear Pin1 signaling. Normally, Pin1 participates in transcriptional regulation and cell survival pathways within the nucleus [30]. Cytoplasmic trapping of Pin1 may therefore contribute to broader neuronal dysfunction beyond tau pathology alone.

##### Phosphorylation-Mediated Inhibition

Pin1 activity and substrate engagement are negatively regulated by inhibitory phosphorylation events, which can impair both substrate recognition and catalytic isomerase activity. Specifically, phosphorylation of Pin1 at Ser16 disrupts substrate recognition by the WW domain, whereas death-associated protein kinase 1 (DAPK1)-mediated phosphorylation at Ser71 suppresses catalytic activity within the PPIase domain [60,62]. Both modifications diminish the efficiency of Pin1-catalyzed cis-trans conversion and shift tau conformational equilibrium toward pathogenic accumulation.

In parallel, hyperactivation of tau kinases such as Cyclin-dependent kinase 5 (CDK5) and glycogen synthase kinase-3β (GSK3β) promotes tau hyperphosphorylation and increases the burden of phosphorylated Pin1 substrates across AD, TBI, stroke, and other neurodegenerative conditions [69,70,71,72]. Together, these kinase-dependent processes can impair the balance between Pin1 activity and pathological tau phosphorylation.

##### Transcriptional Downregulation

Aging and chronic neurodegeneration are associated with a gradual decline in Pin1 expression and function [38,65]. Studies of AD brains reveal substantial reductions in Pin1 mRNA and protein levels in affected cortical and hippocampal regions [61,65]. Age-related alterations in Pin1 localization and function have also been described in association with neuronal lipofuscin accumulation [73].

Several microRNAs identified mainly in cancer and cellular stress contexts, including miR-200b, miR-296-5p, and miR-874, have been shown to negatively regulate Pin1 expression [74,75,76]. Chronic inflammation and cellular stress-related signaling may further reduce Pin1 expression through epigenetic modifications and transcriptional repression mechanisms [77,78].

Reduced Pin1 expression lowers neuronal capacity to maintain tau conformational homeostasis and likely contributes to age-related vulnerability to dementia [38,65]. Because aging represents the strongest risk factor for most tauopathies, chronic Pin1 downregulation may constitute an important initiating event in disease progression.

Collectively, oxidative stress, pathological sequestration, inhibitory phosphorylation, and transcriptional suppression converge to impair Pin1 activity across diverse neurological disorders (Figure 1B). Regardless of the initiating insult, the downstream consequence remains accumulation of pathological cis P-tau and initiation of cistauosis.

#### 2.3.2. Cistauosis: The Early Tauopathy Cascade

The term “cistauosis” was originally proposed as a conceptual framework to describe the early, soluble tau-mediated pathogenic cascade triggered by cis P-tau accumulation prior to irreversible neurodegeneration [14,44]. Unlike classical tauopathy models that emphasize late-stage NFT accumulation, cistauosis highlights early soluble tau-mediated neuronal dysfunction occurring prior to irreversible neurodegeneration [13,14,52].

Cistauosis has been proposed to involve multiple downstream pathological processes associated with cis P-tau accumulation, including axonal injury, mitochondrial dysfunction, synaptic degeneration, neuroinflammation, and prion-like tau propagation [12,13,14,44]. Importantly, these processes precede overt NFT formation, supporting the view that soluble conformationally altered tau acts as an early upstream pathogenic driver.

##### Acute Induction

One defining feature of cistauosis is its rapid induction following neuronal stress [14]. In experimental TBI models, cis P-tau becomes detectable within hours after injury, far earlier than conventional tau aggregates [12,14]. Similar early induction of cis P-tau has been observed following cerebral ischemia and during early-stage AD pathology [23,79].

Acute induction is thought to be closely associated with rapid Pin1 inactivation. Mechanical injury, hypoxia, inflammation, and ROS production have been shown or proposed to impair Pin1 catalytic activity, thereby shifting tau equilibrium toward the cis conformation [14,26]. Because tau phosphorylation at Thr231 also occurs under physiological conditions, failure of Pin1-mediated isomerization may shift normal tau regulation toward pathological cistauosis.

Importantly, cis P-tau induction precedes overt neuronal death, suggesting that early cistauosis is a potentially reversible therapeutic window (Figure 3) [27,44]. Early intervention during this stage may therefore prevent downstream neurodegenerative progression.

##### Axonal Specificity

Early cistauosis is prominently associated with axonal pathology and dystrophic neurites during initial disease stages [12,14]. Long axons are especially dependent on intact microtubule networks for long-distance transport of mitochondria, vesicles, and proteins [34,45]. Because cis P-tau destabilizes microtubules, axonal transport may become progressively impaired [12,14].

An experimental study demonstrated early accumulation of swollen dystrophic neurites following cis P-tau induction [14]. These abnormalities resemble early neuritic and axonal pathology reported in AD and CTE brains [12,13,24].

Mitochondrial transport failure further exacerbates neuronal dysfunction by impairing adenosine triphosphate delivery to synapses [50]. Local energy depletion contributes to synaptic degeneration and ultimately neuronal apoptosis.

The early axonal localization of cis P-tau distinguishes cistauosis from late-stage NFT pathology, which is predominantly somatodendritic [12,14]. This observation supports the hypothesis that axonal dysfunction represents a critical initiating event in tau-mediated neurodegeneration.

##### Prion-like Spread

A defining feature of cistauosis is the progressive propagation of pathological tau across neuronal networks [12,14,25]. Direct evidence for the prion-like propagation of cis P-tau comes from studies in which purified cis P-tau was stereotactically injected into the cortex of wild-type mice, resulting in progressive neurodegeneration, cis P-tau accumulation in brain regions distant from the injection site, and associated axonal and synaptic pathologies. Notably, these pathological and behavioral effects were absent following cis P-tau injection in tau knockout mice, demonstrating that endogenous tau is required for cis P-tau-induced propagation [13]. This study provides direct experimental support for the prion-like propagation of cis P-tau and suggests that pathogenic cis P-tau can propagate tau-dependent pathology beyond the initial site of exposure.

This prion-like mechanism may contribute to the stepwise anatomical spread of tau pathology. In AD, tau pathology progresses from the entorhinal cortex to hippocampal and neocortical regions in highly stereotyped patterns [24,52]. Similar network-based propagation has been reported in CTE and other tauopathies [80].

Exosomes appear to play an important role in tau transmission more broadly [54]. These extracellular vesicles carry pathological tau species between cells and may facilitate long-distance spread through cerebrospinal fluid and circulation.

Importantly, conformation-specific antibodies target not only intracellular but also extracellular cis P-tau, thereby effectively blocking cistauosis propagation in experimental models [13,14]. This finding highlights pathological spread as a therapeutically actionable process.

##### Progressive Neurodegeneration

Persistent cistauosis is proposed to contribute to progressive neurodegeneration characterized by synaptic dysfunction, neuronal loss, gliosis, and eventual NFT formation [12,14]. Over time, soluble cis P-tau oligomers accumulate and may progressively impair cellular clearance pathways, further promoting tau aggregation and neurodegeneration [23,24,25].

Neuroinflammation becomes increasingly prominent during disease progression. Activated microglia and astrocytes release cytokines, complement proteins, and ROS that amplify neuronal injury [81,82]. Chronic inflammation may further impair Pin1 function via oxidative stress, reinforcing pathological feedback loops [26,82].

Eventually, persistent cis P-tau accumulation promotes progressive tau oligomerization and formation of insoluble fibrillar aggregates that are associated with NFT pathology (Figure 3) [24,25,83]. Importantly, accumulating evidence suggests that NFTs represent relatively late downstream consequences of earlier cistauosis rather than the primary initiating toxic species [12,13,14].

This model reframes current understanding of tauopathy progression. Rather than viewing NFTs as the primary drivers of disease, current evidence indicates that early soluble cis P-tau initiates neuronal dysfunction and cistauosis long before the formation of irreversible fibrillar aggregates and NFTs (Figure 3) [12,14,25].

## 3. The Pin1–cis P-tau Axis in Conventional Tauopathies

### 3.1. AD

AD is the most common cause of dementia and is pathologically characterized by extracellular amyloid-β plaques, intracellular NFTs, synaptic degeneration, and progressive neuronal loss [15,84]. Despite decades of intensive research, currently approved therapies provide only limited disease-modifying effects and do not fully halt disease progression [85,86,87].

The classical amyloid cascade hypothesis proposed that amyloid-β accumulation represents the initiating pathogenic event driving downstream tau pathology and neurodegeneration [88]. However, several observations have highlighted limitations of this model and suggest that additional pathogenic mechanisms contribute to disease progression. Cognitive decline correlates more strongly with tau pathology than with amyloid burden [89,90], and anti-amyloid therapies have shown only modest clinical benefits in large-scale trials [87,91].

Compelling evidence suggests that cis P-tau represents an early pathological species in AD [23,24,44]. Rather than merely representing a late secondary consequence of amyloid-β deposition, pathological cis P-tau accumulation appears during early disease stages and may actively contribute to disease progression [24,44].

#### 3.1.1. Pin1 Dysfunction in AD

Pin1 dysfunction has been well documented in AD brains [26,38,63]. Both Pin1 activity and expression are reduced in vulnerable hippocampal and cortical regions. Importantly, these reductions correlate closely with tau pathology severity and cognitive decline [38,65].

Oxidative stress represents a major mechanism underlying Pin1 impairment in AD [26]. Increased ROS production within AD neurons oxidizes catalytic Cys113 and suppresses Pin1 activity [26]. Oxidized and functionally impaired Pin1 accumulates in AD hippocampal regions with severe tau pathology [38,61].

Pin1 association with tau aggregates may further reduce the availability of soluble functional Pin1 in AD brains [31,68]. This process may further exacerbate cis P-tau accumulation by limiting the availability of functional Pin1, potentially creating a pathogenic feed-forward cycle.

Post-translational modifications and aberrant kinase signaling can further impair Pin1 catalytic activity in AD [60,62]. Concurrently, hyperactivation of CDK5 and GSK3β promotes tau hyperphosphorylation and accelerates tau pathology progression [72,92,93]. Together, these processes may synergistically enhance cis P-tau accumulation and downstream neurodegeneration.

Aging-related decline or deficiency of Pin1 may increase neuronal vulnerability to AD-associated pathology [38,65]. Human and experimental studies demonstrate decreased Pin1 levels in neurodegenerative contexts and correlations with tau pathology severity and cognitive impairment [38,65]. Genetic studies further implicate PIN1 promoter polymorphisms and altered expression in increased AD susceptibility [77,94]. Notably, this Pin1 deficit shows sex specificity: reduced PIN1 expression across neocortical and limbic regions correlates with cognitive and neuropathological phenotypes in female, but not male, AD patients [65]. This is of particular interest given that AD disproportionately affects women and that the same axis operates in preeclampsia, a female-specific disorder [95].

Experimental models strongly support a significant role for Pin1 in AD-related neurodegenerative processes. Pin1 knockout mice develop age-dependent tau hyperphosphorylation, synaptic degeneration, and cognitive impairment [38]. However, Pin1 has multiple substrates beyond tau, including proteins involved in APP processing, transcriptional regulation, apoptosis, and inflammatory signaling [96]. Therefore, neurological phenotypes observed following global Pin1 deficiency cannot be attributed exclusively to cis P-tau accumulation.

More specific evidence for a pathogenic contribution of cis P-tau comes from experiments that directly manipulate the cis conformer rather than Pin1 globally. A cis P-tau conformation-specific monoclonal antibody (cis mAb) selectively targets cis P-tau while sparing the trans conformer, and cis mAb treatment attenuates cis P-tau-associated pathological and functional abnormalities in several experimental models [12,13,14]. In addition, administration of purified cis P-tau induces progressive neurodegenerative and behavioral abnormalities in mice, which can be attenuated by cis mAb treatment [13]. These conformation-specific intervention and gain-of-function experiments provide more direct evidence for a pathogenic contribution of cis P-tau than the Pin1 knockout phenotype alone.

Taken together, the available evidence supports a key upstream role for Pin1 dysfunction in pathological tau conformational dysregulation, while the relative contribution of cis P-tau-dependent and other Pin1-dependent mechanisms requires further clarification.

#### 3.1.2. cis P-tau as an Early AD Pathogenic Species

A major advance in understanding tau pathology is the recognition that cis P-tau can accumulate during early stages of AD-related pathology before overt NFT formation [24]. In human brains, cis P-tau is detectable in mild cognitive impairment (MCI) and Braak stage I-II tissues, suggesting that cistauosis may begin before the onset of overt dementia [23,24,44].

Experimental studies demonstrate that cis P-tau accumulates preferentially within dystrophic neurites surrounding amyloid plaques [21,24]. These neuritic abnormalities are characterized by severe microtubule disruption, mitochondrial transport failure, and synaptic degeneration [12,14,44].

cis P-tau may therefore contribute to neurotoxicity traditionally attributed to amyloid-β pathology. Amyloid-associated oxidative and neuronal stress can impair Pin1 activity and facilitate early cis P-tau accumulation, thereby promoting downstream tau-mediated synaptic and neurodegenerative cascades [24,26].

Experimental studies further suggest that pathological cis P-tau can propagate trans-neuronally and seed the pathological conversion of normal tau in neighboring cells through prion-like mechanisms [14,25,83]. However, the extent to which these experimental propagation mechanisms account for the anatomical progression of tau pathology in human AD remains to be established.

#### 3.1.3. Preclinical Potential of cis P-tau-Directed Immunotherapy in AD

The identification of cis P-tau as an early pathogenic conformer has led to the development of cis mAb that selectively target the pathological cis isoform [14,44]. Preclinical studies have provided encouraging evidence that cis P-tau-directed antibodies can modify pathological tau-associated outcomes; however, the interpretation of these findings depends critically on the experimental model used.

In humanized tau (hTau) mice that develop age-related tau pathology and neurodegeneration, cis mAb treatment has been reported to reduce cis P-tau accumulation and limit the progression of neuronal and functional abnormalities [13]. In one preventive paradigm, mice received 300 μg cis mAb intraperitoneally during the first four months of treatment followed by administration every other week, whereas a separate intervention study administered 300 μg intraperitoneally weekly to 13-month-old hTau mice with established tau pathology and memory deficits [13]. These findings provide evidence that targeting cis P-tau can not only prevent the development but also ameliorate the progression of tauopathy, neurodegeneration, and brain dysfunction in a well-established AD tauopathy model.

More recently, a murine antibody (mPNT001) with the same complementarity-determining regions as the humanized cis P-tau mAb (PNT001) was evaluated in female Tg4510 mice expressing mutant human P301L tau [97]. Weekly intraperitoneal administration of 20 or 60 mg/kg mPNT001 from 1.5 to 6.5 months of age improved selected cognitive and synaptic outcomes and reduced several pathological endpoints, including serum neurofilament light chain, neuroinflammatory markers, insoluble tau species, and mature neurofibrillary pathology, particularly at the higher dose [97]. It would be important to evaluate the therapeutic efficacy of cis P-tau-directed antibodies in other AD models including those containing amyloid pathology.

### 3.2. TBI and CTE

TBI affects tens of millions of people each year and is widely recognized as a significant environmental factor increasing the risk of chronic neurodegenerative diseases and dementia [98,99,100]. Recurrent mild traumatic brain injury, especially in athletes and military personnel, is closely associated with the occurrence of CTE [7]. CTE is a progressive neurodegenerative disease characterized by widespread tau protein lesions, cognitive decline, mood and behavioral abnormalities, and motor dysfunction [5]. For many years, the molecular link between acute traumatic brain injury and delayed neurodegeneration lacked clarity largely because overt tau pathology is not readily detectable in human brains after fatal acute brain injury [101]. However, the rapid and robust induction of cis P-tau hours after brain injury provides direct mechanistic evidence for explaining post-traumatic tau protein lesions and subsequent neurodegeneration [12,14].

#### 3.2.1. Acute TBI: Rapid Pin1 Inactivation and cis P-tau Induction

Traumatic brain injury rapidly induces cistauosis, with cis P-tau appearing specifically within damaged axons hours after trauma. This rapid induction precedes detectable NFTs or extensive neuronal loss, indicating that cistauosis represents one of the earliest pathological responses to trauma [12,14].

ROS generated during injury oxidize catalytic Cys113 within Pin1 and suppress isomerase activity [26]. Simultaneously, trauma-associated kinase activation enhances tau phosphorylation at Thr231, increasing substrate availability for pathological cis conversion [12,14,67]. The combination of increased phosphorylation and impaired isomerization strongly favors cis P-tau accumulation.

Axonal pathology develops rapidly following cis P-tau induction. Experimental studies reveal severe microtubule disruption, axonal swelling, impaired mitochondrial trafficking, and synaptic dysfunction shortly after injury [12,14,44]. These abnormalities closely resemble early pathology observed in human TBI brains [12,14].

Importantly, elevated cis P-tau is detectable not only in injured brain tissue but also in CSF and peripheral blood after TBI [12]. Plasma cis P-tau levels correlate with injury severity and may predict long-term neurological outcome [12,102].

Notably, cis P-tau accumulation appears early after traumatic brain injury and precedes overt NFT formation [12,14]. Early post-traumatic pathology is characterized by axonal cis P-tau accumulation and formation of soluble pathogenic tau species, supporting the hypothesis that cistauosis may initiate chronic traumatic neurodegeneration [12,14,25].

Conformation-specific monoclonal antibodies targeting cis P-tau have demonstrated robust neuroprotective effects in experimental models of traumatic brain injury and trauma-associated tauopathy [12,14,44]. In acute injury paradigms, cis mAb administration reduces injury-induced cis P-tau accumulation, preserves axonal integrity, and mitigates structural and functional neuronal damage following injury [12,14].

Another key feature of post-traumatic cistauosis is its persistence. Although many acute injury responses resolve over time, cis P-tau can remain chronically elevated and continue to propagate pathology long after the initial trauma [12,14]. This sustained pathological process may help explain the delayed neurodegenerative consequences observed after TBI.

#### 3.2.2. Chronic TBI/CTE: Persistent cis P-tau Drives Progressive Tauopathy

Repeated TBI is thought to promote cumulative cis P-tau accumulation and progressive neurodegeneration characteristic of CTE [12,14,103]. Postmortem examinations of athletes diagnosed with CTE reveal widespread tau pathology in the cortex and deeper brain structures [5,103], and cis P-tau has been identified within these pathological lesions and implicated as an early pathogenic species [12].

Clinically, chronic tau pathology in CTE correlates with cognitive impairment, mood disorders, impulsivity, depression, and motor dysfunction [5]. Neuropsychiatric manifestations likely reflect progressive dysfunction within vulnerable frontal and limbic brain regions affected by tau pathology.

Persistent propagation of pathogenic tau species likely contributes to progressive neurodegeneration following repetitive brain injury [12,52], which helps to explain why CTE symptoms often emerge years or decades after repetitive head trauma [104]. Initial injury may trigger persistent low-level cistauosis that gradually propagates and accumulates over time, potentially contributing to delayed clinical manifestation [12,14,25].

Experimental models of repetitive mild TBI demonstrate that cis mAb intervention can significantly suppress long-term tau pathology progression and reduce chronic neurodegenerative burden months after injury [12]. These findings are consistent with broader evidence supporting prion-like tau propagation as a driver of progressive pathological spread within connected neural networks [52,53].

Importantly, TBI is associated with increased long-term risk of AD and related dementias [98,99,105], and persistent cis P-tau pathology provides a potential mechanistic link between brain injury and accelerated neurodegenerative aging [12,14].

Collectively, these findings support the concept that cis P-tau acts as an early and functionally significant mediator of trauma-induced neurodegeneration. Targeting pathological tau conformers therefore represents a promising disease-modifying strategy for TBI- and CTE-associated neurodegenerative disorders [12,14,44].

### 3.3. Evidence and Knowledge Gaps in Other Primary Tauopathies

Whether the Pin1–cis P-tau axis contributes broadly to other primary tauopathies remains insufficiently defined. Early neuropathological studies reported altered Pin1 distribution and its association with conformationally altered pThr231 tau in Pick’s disease and frontotemporal dementia and parkinsonism linked to chromosome 17 (FTDP-17), suggesting that Pin1 dysregulation may participate in selected non-AD tauopathies. However, these studies did not employ the currently available cis conformation-specific antibodies and therefore do not directly establish the presence of cis P-tau or cistauosis. In the same study, Pin1 immunoreactivity was sparse and rarely colocalized with conformationally altered pThr231 tau in progressive supranuclear palsy (PSP), suggesting that Pin1-related mechanisms may differ substantially among tauopathies [68].

More recently, a comparative analysis of human tauopathy brains reported increased cis pThr231-Pro232 tau in AD and argyrophilic grain disease (AGD), while demonstrating distinct tau phosphorylation profiles across PSP, corticobasal degeneration (CBD), and Pick’s disease [106]. Direct evidence linking Pin1 dysfunction to cis P-tau accumulation or demonstrating a causal role for cistauosis in PSP, CBD, Pick’s disease, or primary age-related tauopathy (PART) is currently lacking. Future studies using validated conformation-specific assays and disease-relevant models will be required to determine whether cis P-tau represents a shared pathogenic mechanism or a disease-selective pathway.

## 4. The Pin1–cis P-tau Axis in Unconventional Tauopathies

### 4.1. Ischemic Stroke and VaD

Ischemic stroke is one of the leading causes of disability and cognitive impairment worldwide [107]. Stroke survivors frequently develop long-term vascular cognitive impairment and dementia, yet the molecular mechanisms linking acute ischemic injury to chronic neurodegeneration remain incompletely understood [9,108,109].

Recent evidence suggests that dysregulation of the Pin1–cis P-tau axis may represent one mechanism linking cerebral ischemia to progressive vascular neurodegeneration (Figure 2) [13,27]. Ischemic and hypoperfusion-associated stress may disrupt normal Pin1-mediated tau isomerization following cerebrovascular injury, primarily through oxidative inactivation of Pin1 and enhanced tau phosphorylation [13,26]. Beyond oxidative inactivation, ischemic and hypoperfusion stress engages a specific kinase-driven route to Pin1 loss. DAPK1 phosphorylates Pin1 and inhibits its prolyl isomerase activity [62], and DAPK1 activation after cerebral injury drives cis P-tau accumulation and neurodegeneration, whereas DAPK1 inhibition attenuates both [67]. Parallel activation of CDK5, through calpain-mediated conversion of p35 to p25, further promotes tau hyperphosphorylation in both hemispheres after stroke [71]. Together, these pathways provide a defined molecular link between an ischemic insult and the loss of Pin1-mediated tau regulation that initiates cistauosis [13,26]. Chronic cerebral hypoperfusion is a major contributor to vascular cognitive impairment and may create conditions favorable for cistauosis-associated neurodegeneration [13,109,110]. Sustained reductions in cerebral blood flow induce hypoxia-associated mitochondrial dysfunction and oxidative stress, conditions that may impair Pin1 activity and favor persistent cis P-tau accumulation and progressive vascular tauopathy [13,26,27]. In parallel, inflammatory activation may further amplify neuronal injury and facilitate pathological tau propagation [53,82].

In experimental models of cerebral ischemia, cis P-tau rapidly accumulates in the peri-infarct region and progressively increases over time, preceding widespread neuronal death [79]. In these preclinical settings, conformation-specific anti-cis P-tau therapy has shown neuroprotective effects in experimental ischemic injury, including improved neuronal survival, preserved axonal integrity, and enhanced functional recovery [12,14,111]. The most direct evidence linking the Pin1–cis P-tau axis to VaD comes from a study combining human VaD brain samples and the bilateral common carotid artery stenosis (BCAS) mouse model [13], which has been widely used to mimic key aspects of vascular cognitive impairment and dementia [112,113,114,115]. Robust cis P-tau accumulation was detected in all post-mortem VaD brain samples examined in the absence of the NFTs that define classical tauopathies [13]. In mice, chronic cerebral hypoperfusion induced by BCAS reproduces aspects of this pathology and produces executive-function deficits [112,113,114,115]. In this model, conformation-specific anti-cis P-tau antibody treatment, brain-specific Pin1 restoration, and DAPK1 depletion each rescue the cis P-tau burden and improve cognitive phenotypes, providing mechanistic evidence that the Pin1–cis P-tau axis contributes to vascular cognitive impairment [13]. Moreover, purified soluble cis P-tau is by itself sufficient to induce VaD-like neurodegeneration and brain dysfunction, and single-cell transcriptomic profiling shows that young hypoperfused mice acquire a cortical gene-expression signature resembling those of BCAS mouse brains and early human AD brains, changes largely reversed by anti-cis P-tau immunotherapy [13]. These findings support the Pin1–cis P-tau axis as a potentially targetable pathway in experimental vascular cognitive impairment, while its contribution to post-stroke neurodegeneration in humans requires further investigation.

The detection of cis P-tau in human VaD brain samples, together with mechanistic findings from experimental chronic hypoperfusion models, raises the possibility that cis P-tau contributes to a subset of VaD characterized by pathological tau conformation dysregulation [13,27]. Future studies in larger and clinically well-characterized cohorts will be required to determine the prevalence of cis P-tau pathology and whether a distinct cis P-tau-positive subgroup of VaD can be defined.

### 4.2. PE: Acute cis P-tau Pathology and Potential Long-Term Implications

PE is a multisystem pregnancy disorder characterized by new-onset hypertension, endothelial dysfunction, placental ischemia, oxidative stress, and systemic inflammation after 20 weeks of gestation, affecting approximately 3–8% of pregnancies worldwide [116,117]. Beyond the acute maternal and fetal complications, a growing body of epidemiological work indicates that a history of PE carries a substantially increased long-term risk of dementia, and that this risk falls disproportionately on the vascular subtype. In a Danish nationwide cohort, women who developed PE had a more than threefold higher risk of later vascular dementia, an association that strengthened with age at diagnosis and was largely specific to vascular dementia rather than to AD [118]. A subsequent meta-analysis of hypertensive disorders of pregnancy reinforced this pattern, estimating roughly a 1.4-fold higher risk of all-cause dementia and a 1.6-fold higher risk of vascular dementia in women with prior PE [119]. The offspring of PE pregnancies are likewise affected, showing elevated rates of neurodevelopmental impairment, cerebrovascular abnormality, and neuropsychiatric disorder [120,121]. The vascular predominance of the maternal risk raises the possibility that vascular mechanisms may contribute to the long-term neurological consequences associated with PE. However, the molecular pathways linking an acute pregnancy disorder to dementia decades later remain unknown.

The Pin1–cis P-tau axis has been demonstrated to be a major mechanism contributing to the acute vascular and placental abnormalities of PE [11,27]. Whether this pathway also contributes to the elevated long-term dementia risk observed epidemiologically after PE remains unclear. Placental trophoblasts sustain a high metabolic demand and are acutely sensitive to oxidative injury; in PE, placental hypoxia inactivates Pin1, predominantly through oxidation of its catalytic Cys113 residue—and thereby drives the accumulation of cis P-tau within the placenta itself [11,26]. Significant cis P-tau is present in both the placental tissue and the serum of PE patients, and the same induction is reproduced in primary human trophoblasts exposed to hypoxia or to serum from PE patients, confirming that the trigger is carried in the maternal circulation [11]. Critically, cis P-tau is not induced in isolation. It appears together with the anti-angiogenic factors soluble fms-like tyrosine kinase-1 (sFlt-1) and soluble endoglin (sEng), and its accumulation coincides with disrupted trophoblast invasion and impaired endovascular remodeling, the defective placentation that lies at the heart of PE [11,122]. The axis therefore operates upstream of the recognized angiogenic imbalance of the disease rather than merely alongside it.

Because the placenta releases cis P-tau into the maternal circulation, its consequences become systemic, a state that has been termed systemic cistauosis to distinguish it from the neuronal cistauosis in brain disorders [59]. Circulating cis P-tau has been implicated experimentally in endothelial and vascular dysfunction associated with PE [11,27]. It would be interesting to determine whether placental or circulating cis P-tau persists after pregnancy, enters the maternal brain, or mediates the increased dementia risk observed decades later.

A second, distinct dimension of PE-associated cistauosis concerns the developing fetus. Because fetal neurons possess relatively immature antioxidant and proteostatic defenses, the fetal brain may be especially vulnerable to stress-induced disruption of tau homeostasis [123,124]. Maternal inflammation, oxidative stress, and placental dysfunction during PE are associated with impaired neuronal maturation, cerebrovascular abnormality, and enhanced tau phosphorylation in the offspring [120,125]. Direct evidence that maternal cis P-tau propagates into the fetal compartment remains limited, and this distinction should be preserved; what is established is that prenatal hypoxia and gestational stress predispose to neurodevelopmental and neurodegenerative disorders in later life [123,124]. These observations are consistent with a developmental-origins model in which early-life vascular and inflammatory insults prime neural circuits for vulnerability that only becomes manifest much later, and they suggest that the reach of the Pin1–cis P-tau axis in PE may extend across two generations.

The strongest mechanistic evidence for a role of cis P-tau in the acute pathophysiology of PE comes from antibody-based depletion experiments [11]. Removing cis P-tau from PE patient serum with the conformation-specific antibody almost entirely abolishes the ability of that serum to disrupt trophoblast invasion and endovascular activity in vitro [11]. The same manipulation is decisive in vivo: injected into pregnant humanized-tau mice, serum from PE patients reproduces the full syndrome—hypertension, proteinuria, placental protein aggregation, glomerular endotheliosis, and fetal growth restriction— whereas serum depleted of cis P-tau does not, and treatment with the antibody corrects these features and restores a normal pregnancy [11]. Together, these experiments provide mechanistic evidence that circulating cis P-tau contributes to PE-like placental and systemic abnormalities in the experimental settings examined. They also support the concept of PE as a proposed unconventional systemic tauopathy, in which a disease-associated tau conformer generated outside the brain may contribute to placental and vascular dysfunction (Figure 2). It would be interesting to examine whether PE-associated cis P-tau causes subsequent maternal neurodegeneration or dementia.

## 5. cis P-tau as a Potential Early Diagnostic and Prognostic Biomarker

One of the major translational implications of the Pin1–cis P-tau axis is the potential of cis P-tau as an early biomarker across neurodegenerative, traumatic, and vascular tau-related disorders [11,12,23,24]. Plasma p-tau217 has emerged as a leading blood biomarker for early AD, detecting preclinical pathology with high accuracy and outperforming p-tau181 and neurofilament light chain [126]. Its clinical standing was underscored in May 2025, when the U.S. Food and Drug Administration (FDA) cleared the first blood test for AD diagnosis—the Lumipulse G pTau217/β-Amyloid 1-42 Plasma Ratio—for detection of amyloid pathology in symptomatic adults [127]. However, p-tau217 and related phospho-tau assays quantify phosphorylation without resolving the underlying peptide-bond conformation and therefore do not distinguish cis from trans tau conformers. This distinction is particularly important for p-tau231, because phosphorylation at Thr231 and adoption of the cis conformation at the pThr231-Pro motif represent related but distinct molecular features. General p-tau231 assays detect phosphorylated tau species containing or associated with the Thr231 epitope and should not be considered measurements of cis P-tau unless conformational selectivity for the cis pThr231-Pro isomer has been specifically demonstrated. cis P-tau, by contrast, is a conformation-specific pathogenic tau species that emerges early, before overt NFT accumulation and clinical manifestations, across several experimental and clinical settings [14,23,24,28,43]. Accordingly, cis P-tau could add conformational and mechanistic resolution to existing phospho-tau biomarker frameworks.

The distinctive feature of cis P-tau lies in its conformational specificity. Whereas conventional phospho-tau assays detect tau phosphorylation regardless of whether the relevant peptide bond adopts a physiological or pathological conformation, cis-specific antibodies selectively recognize the pathogenic cis isomer of pThr231-Pro tau while sparing the physiological trans conformation [23,24,43]. This conformational selectivity provides additional molecular resolution by distinguishing a disease-associated tau conformer from other phosphorylated tau species. However, whether this property translates into greater diagnostic specificity and sensitivity than established plasma biomarkers such as p-tau217 has not yet been demonstrated in direct clinical comparisons.

In AD, neuropathological studies have demonstrated that cis P-tau accumulates during prodromal stages, including MCI and Braak stage I/II pathology, and elevated cis P-tau has also been detected in CSF samples from patients with AD, supporting its potential utility as an early biomarker [23,24]. Separately, plasma p-tau231 has been reported to become abnormal before amyloid PET reaches the conventional positivity threshold, supporting p-tau231 as an early marker along the AD continuum [128]. However, this assay did not quantitatively distinguish native cis from trans pThr231 tau. Therefore, these findings should be interpreted as evidence for early changes in plasma p-tau231 rather than as direct evidence for circulating cis P-tau [128]. Experimental studies suggest that cis P-tau accumulation contributes to neuronal and synaptic dysfunction, progressive neurodegeneration, and functional impairment [12,14]. Because cis P-tau appears long before extensive NFT accumulation, it may provide opportunities for early therapeutic intervention [23,24].

The biomarker potential of cis P-tau extends beyond AD into traumatic and vascular insults and neurodegeneration. Following acute TBI, cis P-tau is rapidly induced not only in injured cortical axons, but also detected in cerebrospinal fluid, with its levels associated with traumatic axonal injury, injury severity, injury frequency, and long-term functional outcomes in human patients and animal models [12,14,102]. Persistent cis P-tau accumulation following repetitive brain injury has been shown to cause chronic neurodegenerative sequelae and CTE-related tau pathology, which are rescued by eliminating cis P-tau using cis antibody immunotherapy [12,14]. These findings are particularly relevant because current TBI biomarkers remain limited in their ability to predict chronic neurodegeneration after mild or repetitive brain injury [7,129].

Similarly, cis P-tau accumulation has been reported in human VaD and experimental models of ischemic or hypoperfusion-associated brain injury [13,79]. These findings support further investigation of cis P-tau as a candidate biomarker in vascular cognitive impairment, including whether it may identify a biologically distinct cis P-tau-positive subgroup. Stroke-related brain injury, including infarction and white matter damage, is itself a major contributor to long-term vascular cognitive decline [8,109]. cis P-tau accumulates rapidly following ischemia in experimental models, supporting further investigation of this conformer as a candidate biomarker of post-ischemic neurodegeneration [79]. In addition, recent antibody-intervention studies support cis P-tau as a potential therapeutic target in experimental cerebral ischemia [111].

In PE, elevated cis P-tau has been detected in maternal serum and placental tissue, and experimental immunodepletion studies support a contribution of circulating cis P-tau to acute PE-like pathology [11,59]. These findings support further investigation of cis P-tau as a biomarker of PE-associated pathology. It would be interesting to determine its value for predicting subsequent maternal dementia or offspring neurological outcomes. Together, these findings suggest that the potential biomarker utility of cis P-tau may extend beyond classical neurodegenerative disorders, although its clinical significance will require disease-specific validation.

Blood-based tau biomarkers offer substantial clinical advantages over CSF sampling and neuroimaging because they are minimally invasive, scalable, and well suited for longitudinal monitoring and population screening [130,131,132]. In parallel, advances in ultrasensitive analytical platforms, including single-molecule array (Simoa), immunoprecipitation coupled with mass spectrometry detection, and immunoprecipitation coupled with next-generation DNA-based signal amplification technologies, have markedly improved the detection of low-abundance pathological tau species in biological fluids [128,131,133]. It would be critical to develop ultrasensitive technologies to quantify conformation-specific species such as cis P-tau because they may be present at very low concentrations during the earliest stages of disease.

Several challenges nevertheless remain before routine clinical implementation can be achieved. First, assay standardization is required to establish analytical reproducibility, reference ranges, and clinically validated cutoff values [130,134]. Second, large multicenter longitudinal studies, including direct head-to-head comparisons with established biomarkers such as p-tau217, are necessary to define the diagnostic and prognostic performance of conformation-specific cis P-tau assays and determine whether they provide incremental clinical value [131]. Third, the relationship between circulating cis P-tau levels and tissue pathology requires further clarification, particularly in systemic diseases such as PE [11]. Finally, blood-based biomarker measurements can be influenced by pre-analytical variables such as sample collection, processing, storage, and freeze–thaw conditions [135]. These considerations may be particularly important for cis P-tau-specific assays, for which preservation of conformational integrity will require careful validation. Rigorous standardization of these variables will therefore be important before cis P-tau can be developed as a robust clinical or companion diagnostic [131,136].

Despite these technical and translational challenges, available findings support further evaluation of cis P-tau as an emerging biomarker candidate for early disease detection, risk stratification, prognosis, and therapeutic monitoring. However, its clinical performance and utility are likely to differ among disease contexts and require disease-specific validation in independent longitudinal cohorts.

## 6. Clinical Translation, Challenges and Future Perspectives

The discovery of the Pin1–cis P-tau axis has redirected therapeutic efforts from late-stage tau aggregates toward the early pathogenic conformer that initiates them [14,23,24]. Conformation-specific cis P-tau monoclonal antibody therapy represents one of the most advanced translational applications of this paradigm and has shown efficacy across both conventional and unconventional experimental tauopathy models [13,14].

### 6.1. Clinical Development of cis P-tau-Directed Immunotherapy

Conformation-specific targeting of cis P-tau provides a therapeutic strategy designed to distinguish a disease-associated tau conformer from phosphorylated tau species that retain physiological functions. Preclinical studies using cis P-tau-directed antibodies have reported beneficial effects across several experimental contexts, providing a strong rationale for further translational development (Table 2).

Several related versions of the conformation-specific antibody targeting cis pThr231 tau, derived from the original cis mAb [14,24], have been used across mechanistic, preclinical, and clinical studies with generally consistent findings. In this review, “cis mAb” refers to the experimental antibody used in mechanistic and preclinical intervention studies [12,13,14], whereas mPNT001 and its humanized counterpart PNT001 refer to the related antibodies evaluated in Tg4510 mice and human studies, respectively [97,137,138]. Mechanistic studies using experimental cis mAb have implicated extracellular neutralization and intracellular TRIM21-dependent proteasomal degradation in cis P-tau clearance [12,14]; however, these mechanisms have not yet been specifically established for mPNT001 or PNT001.

In the context of AD-related tau pathology, the available therapeutic evidence derives primarily from experimental tauopathy models. In hTau mice, in which endogenous mouse tau is replaced by human tau and age-dependent tau pathology develops, cis mAb treatment beginning at 3 months of age and continuing for 8 months reduced cis P-tau accumulation, attenuated NFT-like pathology, and prevented learning and memory deficits [13]. In a separate intervention paradigm, 13-month-old hTau mice with pre-existing tau pathology and cognitive deficits received cis mAb for 6 months; treatment eliminated cis P-tau, attenuated further neuronal loss, and improved working-memory performance, although established NFT-like pathology was not significantly reduced [13]. More recently, mPNT001, a murine surrogate carrying the same complementarity-determining regions as PNT001, was evaluated in female Tg4510 mice expressing mutant human P301L tau. Animals received 20 or 60 mg/kg mPNT001 intraperitoneally once weekly from approximately 1.5 to 6.5 months of age (23 total doses), with improvements in selected spatial-memory and synaptic-plasticity measures and reductions in several biochemical and pathological endpoints, particularly at the higher dose [97].

These findings provide proof-of-concept that cis P-tau-directed immunotherapy can prevent or ameliorate tau-associated pathological and functional abnormalities in selected experimental tauopathy models. Because hTau and Tg4510 mice primarily model tau-related pathology rather than the combined amyloid–tau pathological spectrum of sporadic AD, further evaluation in additional AD models incorporating amyloid pathology, such as APP/PS1 and 3xTg mice, will be important to define the broader therapeutic potential of cis P-tau-targeted immunotherapy in AD.

Additional mechanistic evidence comes from a purified cis P-tau injection paradigm in wild-type mice. Stereotactic cortical administration of purified soluble cis P-tau produced progressive spreading of cis P-tau, neurodegenerative changes, and behavioral abnormalities, whereas cis mAb treatment markedly attenuated these effects [13]. This model provides additional evidence that cis P-tau itself can propagate pathology and that its neutralization can modify this process [13].

In contrast, the therapeutic evidence in experimental TBI is based on disease-specific injury models. In single severe TBI mice, cis mAb was tested in both acute and delayed-treatment paradigms. Albayram et al. administered 200 μg cis mAb intraperitoneally in short-course regimens consisting of three or four injections over 10 days; treatment remained effective when initiation was delayed by 4–8 h after injury [12]. Longer intermittent treatment for 4 months also attenuated chronic cis P-tau accumulation, tau-associated pathology, and functional abnormalities. In repetitive mild TBI, seven injuries were delivered over 9 days, followed by cis mAb treatment over 4 months and a 2-month washout; treatment reduced CTE-like pathological and behavioral abnormalities [12]. These findings extend the original demonstration by Kondo et al. that cis mAb treatment after experimental TBI blocks early cistauosis and subsequent tauopathy [14].

Therapeutic effects have also been demonstrated in the BCAS model of vascular cognitive impairment. Two-month-old mice received 300 μg cis mAb intraperitoneally every 3 days for four doses, followed by 150 μg weekly for the 28-day paradigm; in the longer 6-month paradigm, the initial loading regimen was followed by 200 μg weekly [13]. Treatment reduced cis P-tau accumulation, neuroinflammatory and white-matter abnormalities, and cognitive dysfunction despite persistent cerebral hypoperfusion [13]. These findings demonstrate therapeutic activity of cis P-tau-targeted immunotherapy in a chronic vascular-insufficiency model.

The humanized antibody PNT001 has undergone early-phase clinical evaluation. In a randomized, double-blind, placebo-controlled Phase 1 study, healthy volunteers received single intravenous doses ranging from 33 to 4000 mg, and PNT001 was generally well tolerated with dose-related serum and CSF exposure [137]. At doses of 900 mg and above, CSF concentrations exceeded the measured binding-affinity constant for cis-pT231 tau, demonstrating CNS exposure [137]. A multiple-ascending-dose Phase 1 study has also evaluated PNT001 in hospitalized patients with acute TBI [138]. Further clinical studies will be important to determine the therapeutic efficacy of PNT001 in AD and other tau-related disorders.

### 6.2. cis P-tau Targeting in the Broader Landscape of Tau-Directed Therapies

Tau-directed therapeutic strategies encompass a broad range of approaches aimed at reducing pathological tau burden, limiting aggregation or propagation, or selectively targeting disease-associated tau species [139]. Because tau contributes to normal neuronal functions, including microtubule regulation, complete or prolonged suppression of tau may also carry potential functional consequences, as suggested by neurological abnormalities observed in tau knockout models [140,141]. Accordingly, current tau-directed strategies seek to achieve an appropriate balance between reducing pathological tau and preserving physiological tau function.

Moreover, multiple antibodies directed at total or phosphorylated tau irrespective of conformation have repeatedly failed to demonstrate clear disease-modifying efficacy in Phase 2 trials [142,143,144]. Although these clinical failures likely reflect multiple factors, the inability to selectively distinguish potentially pathogenic tau species from tau conformers with physiological functions may represent one limitation of such approaches. By contrast, cis mAb preferentially targets the pathological cis conformer while sparing trans P-tau [14,43], providing a therapeutic strategy based on the conformational biology of the Pin1–cis P-tau switch rather than on tau phosphorylation alone.

This mechanistic selectivity may also help explain the breadth of the reported preclinical effects. cis P-tau has been identified as a downstream consequence of impaired Pin1-dependent tau regulation in neurodegenerative, traumatic, and vascular cognitive disorders [13,14,27], raising the possibility that selective targeting of this conformer may be therapeutically relevant across conditions in which this pathway is activated. Consistent with this concept, cis P-tau-directed antibodies have shown beneficial effects across acute and chronic pathological outcomes in experimental models of TBI, cerebral ischemia or vascular cognitive impairment, and PE [11,13,14], in addition to AD-related tauopathy models. Because TBI, cerebrovascular disease, and PE have each been associated with an increased risk of subsequent cognitive impairment or dementia [98,99,108,118], these findings raise the possibility that cis-targeted therapy could have utility across multiple disease contexts. However, whether targeting cis P-tau in these conditions can alter long-term dementia risk remains to be established clinically.

### 6.3. Translational Challenges

Several obstacles remain. The first is delivery across the blood–brain barrier (BBB). Although antibody access to injured or inflamed brain may be enhanced by localized BBB disruption, achieving sufficient and sustained CNS exposure in chronic neurodegenerative settings is difficult [142,145]. Encouragingly, Phase 1 data showed dose-related PNT001 exposure in the CSF, with concentrations at doses ≥900 mg exceeding the measured binding-affinity constant for cis-pT231 tau [137]. These pharmacokinetic findings support CNS exposure, but do not by themselves establish target engagement or therapeutic efficacy. In addition, delivery strategies under investigation—receptor-mediated transcytosis, Fc engineering, intranasal administration, focused ultrasound-mediated BBB opening, and nanoparticle-based carriers—may further improve brain exposure [146,147,148,149].

A second issue is long-term safety. Because tau contributes to microtubule stabilization and synaptic plasticity, prolonged modulation of tau pathways could carry unforeseen neurophysiological consequences [44,142]. The cis-selective design mitigates this risk by sparing trans P-tau or other normal tau species, but extended clinical monitoring of immunogenicity, potential neurotoxicity, and systemic effects will be required.

A third challenge concerns the assay itself. Because cis P-tau is defined by conformation rather than by phosphorylation alone, its measurement depends on preserving the cis structure, and standardized, conformation-preserving protocols will be needed before a cis P-tau test can complement established phospho-tau biomarkers such as plasma p-tau217 in clinical practice [131,136].

### 6.4. Future Directions

Patient stratification is a central priority. Because cis P-tau appears during early disease stages, therapeutic efficacy will depend heavily on early, biomarker-guided intervention [44,136]. Integrating cis P-tau assays with neuroimaging, genetic profiling, and cognitive testing could enable precision-medicine approaches tailored to individual disease trajectories. Defined-onset conditions such as acute TBI, stroke, or PE may provide particularly informative settings for evaluating the temporal relationship between cis P-tau induction, biomarker changes, and subsequent neurological outcomes (Figure 3) [12,102].

Pharmacological restoration of Pin1 activity offers a complementary but mechanistically broader strategy. Because Pin1 catalyzes conversion of cis P-tau to the trans conformation, restoring Pin1 function could suppress cis P-tau accumulation at an even earlier, upstream point [30,31,60]; consistent with this, brain-specific Pin1 restoration in postnatal neurons rescues the phenotype in experimental VaD [13]. However, these effects cannot be assumed to result exclusively from cis P-tau reduction because Pin1 has other substrates and functions, particularly because Pin1-regulated cell-cycle and oncogenic pathways are also implicated in tumorigenesis [150]. Thus, therapeutic modulation of Pin1 may have substantially broader biological effects than selective targeting of cis P-tau and will require careful assessment of both efficacy and safety.

The scope of the axis may also extend beyond the disorders reviewed here. Emerging evidence implicates tau dysregulation in Parkinson’s disease, ALS, frontotemporal dementia, and aging-associated cognitive decline [151,152]; whether cistauosis specifically contributes to these conditions is an important open question.

Finally, advances in biosensors, ultrasensitive detection, and AI-assisted biomarker analysis may enable large-scale, minimally invasive screening for neurodegenerative disease risk [136,153,154], supporting a shift from late-stage symptomatic treatment toward mechanism-based precision medicine. Taken together, the available findings provide a rationale for further evaluating cis P-tau as a biomarker and therapeutic target in appropriately selected disease contexts. Prospective longitudinal studies and controlled clinical trials will be required to determine whether early detection or therapeutic targeting of cis P-tau can improve long-term neurological outcomes.

## 7. Conclusions

The Pin1–cis P-tau axis reframes tauopathy as a process that, among others, begins with early conformational dysregulation of phosphorylated tau. In this framework, loss or impairment of Pin1 can favor accumulation of cis P-tau, which has been associated with tau dysregulation, neuroinflammation, and pathological propagation. However, the strength of evidence for this pathway differs substantially among diseases. Mechanistic and interventional evidence is particularly strong in AD-related tauopathy, TBI and vascular cognitive impairment models. In PE, cis P-tau has been implicated in acute placental and systemic pathology, but a mechanistic link to long-term maternal dementia has not been established. The Pin1–cis P-tau axis thereby provides a potential convergent molecular mechanism contributing to both conventional and unconventional tauopathies associated with neurodegenerative, traumatic, and vascular insults.

cis P-tau represents a promising candidate biomarker for early diagnosis, prognostic assessment, and therapeutic monitoring. As a relatively early pathogenic tau conformer, cis P-tau may enable minimally invasive detection strategies and earlier intervention before irreversible neurodegeneration develops. Emerging fluid biomarker and biosensor-based approaches further support the translational potential of cis P-tau for disease stratification and longitudinal monitoring across diverse tauopathies.

Conformation-specific cis P-tau antibody therapy provides encouraging preclinical proof-of-concept for disease-modifying intervention directed against the early tau pathology. By selectively targeting pathogenic cis P-tau while preserving other tau conformations, conformation-specific antibodies represent a potential therapeutic strategy across selected tau-related disorders. However, the strength of the supporting evidence varies among disease contexts, and further independent preclinical validation and clinical studies will be required to define their therapeutic scope.

In summary, the Pin1–cis P-tau axis provides an integrated framework for understanding, diagnosing, and potentially treating a broad spectrum of conventional and unconventional tauopathies, among others. As understanding of pathogenic tau conformations continues to evolve, targeting the Pin1–cis P-tau axis may help shift the field from late-stage symptomatic management toward mechanism-based precision medicine focused on earlier detection, prevention, and disease modification.

## Figures and Tables

**Table 1:** Major differences between cis and trans P-tau properties.

Property	trans P-tau	cis P-tau	References
Predominant functional association	Associated with physiological tau function and homeostasis	Associated with pathological tau changes under disease-related or stress conditions	[24,43]
Relationship to Pin1	Generated or maintained by Pin1-mediated isomerization	Accumulate when Pin1 activity is impaired	[31,33]
Microtubule interaction	Retains greater compatibility with microtubule-associated functions	Associated with reduced microtubule affinity and cytoskeletal disruption in experimental models	[24,31,43]
Dephosphorylation and degradation	Prone to dephosphorylation by protein phosphatase 2A (PP2A) and degradation by proteasome	Resistant to dephosphorylation by PP2A and degradation by proteasome	[24,33]
Proteostasis/turnover	Compatible with physiological turnover and clearance	Prone to accumulation and impaired clearance	[23,24,33]
Neurotoxicity	Not associated with overt neurotoxicity under physiological conditions	Induces axonal injury, mitochondrial impairment, synaptic dysfunction, and neuronal loss	[12,14,24]
Inflammatory response	Minimal pro-inflammatory activity	Associated with microglial/astrocytic activation and neuroinflammation	[12,13,14]
Propagation/seeding	Limited aggregation or propagation potential	Demonstrates prion-like propagation and seeding potential	[14,24,25]
Therapeutic relevance	Selectively targeted by trans conformation-specific antibodies	Selectively targeted by cis conformation-specific antibodies	[14,44]

**Table 2:** Representative studies evaluating cis P-tau-directed antibody strategies across experimental and clinical settings.

Disease Context	Experimental Model	Antibody/Intervention	Treatment or Experimental Paradigm	Main Findings	References
Transgenic tauopathy	Female Tg4510 mice expressing mutant human P301L tau	mPNT001	20 or 60 mg/kg, i.p., once weekly from ~1.5 to 6.5 months of age; 23 total doses	Improved selected spatial-memory and LTP outcomes and reduced NfL, neuroinflammatory markers, insoluble tau species, and mature tau pathology, particularly at 60 mg/kg	[97]
Clinical development	Healthy volunteers	PNT001	Single IV doses, 33–4000 mg	Generally well tolerated; dose-related serum and CSF exposure	[137]
Clinical development—acute TBI	Hospitalized acute TBI patients	PNT001	Multiple-ascending-dose Phase 1 study	Registered Phase 1 clinical study	[138]
AD-related experimental tauopathy, prevention	hTau mice expressing human tau on a mouse tau-null background; treatment initiated at 3 months of age	cis mAb	300 μg/mouse, i.p.; treatment for 8 months, with more frequent administration during the first 4 months followed by every-other-week dosing	Reduced cis P-tau, attenuated NFT-like pathology, and prevented learning and memory deficits	[13]
AD-related experimental tauopathy, established pathology	13-month-old hTau mice with pre-existing tau pathology and cognitive deficits	cis mAb	300 μg/mouse, i.p., weekly for 6 months	Eliminated cis P-tau, attenuated further neuronal loss, and improved working-memory measures; established NFT-like pathology was not significantly reduced	[13]
Experimental tau propagation	Purified soluble cis P-tau stereotactically injected bilaterally into the cortex of 3-month-old WT mice	cis mAb	cis mAb administered during the post-injection follow-up paradigm; pathological and behavioral outcomes assessed at 1 and 10 months	Prevented cis P-tau-induced progressive spreading, neurodegenerative changes, synaptic dysfunction, and behavioral abnormalities	[13]
Vascular cognitive impairment, short-term	BCAS in 2-month-old WT mice	cis mAb	300 μg/mouse, i.p., every 3 days for four doses, followed by 150 μg weekly; treatment continued for 28 days	Reduced cis P-tau, neuroinflammation, and demyelination and improved synaptic and cognitive outcomes	[13]
Vascular cognitive impairment, long-term	BCAS in 2-month-old WT mice	cis mAb	300 μg/mouse, i.p., every 3 days for four doses, followed by 200 μg weekly for up to 6 months	Reduced widespread cis P-tau-associated pathology and improved long-term behavioral outcomes despite chronic hypoperfusion	[13]
Single severe TBI, acute/delayed intervention	Weight-drop closed-head ssTBI in young adult C57BL/6 mice	cis mAb	200 μg/injection, i.p.; either four doses on days 1, 3, 7, and 10 or three doses on days 1, 3, and 5; treatment initiation tested immediately and with delays of up to 4–8 h after injury	Reduced cis P-tau and acute axonal pathology and improved sensorimotor/coordination outcomes	[12]
Single severe TBI, chronic intervention	Weight-drop closed-head ssTBI in C57BL/6 mice	cis mAb	Intermittent systemic cis mAb treatment for 4 months followed by a 2-month washout	Reduced chronic cis P-tau spreading, axonal and tau-associated pathology, and several long-term functional deficits	[12,14]
Repetitive mild TBI/CTE-like pathology	Seven closed-head mild TBI events over 9 days in mice	cis mAb	Repeated systemic treatment over 4 months followed by a 2-month washout	Reduced cis P-tau spreading and attenuated CTE-like tau pathology, neuroinflammation, neurodegeneration, and functional abnormalities	[12]
Preeclampsia-associated pathology	Pregnant humanized tau mice receiving serum from patients with preeclampsia	cis P-tau immunodepletion	Patient serum was depleted of cis P-tau before administration to pregnant mice	Attenuated PE-like placental, vascular, renal, maternal, and fetal abnormalities induced by patient serum	[11]

Abbreviations: AD, Alzheimer’s disease; BCAS, bilateral carotid artery stenosis; CSF, cerebrospinal fluid; CTE, chronic traumatic encephalopathy; hTau, humanized tau; i.p., intraperitoneal; LTP, long-term potentiation; NfL, neurofilament light chain; ssTBI, single severe traumatic brain injury; TBI, traumatic brain injury; WT, wild type.

## Data Availability

No new data were created or analyzed in this study. Data sharing is not applicable to this article.
